# Accuracy of Stereophotogrammetry Technique versus Intraoral Scanners for Complete-Arch Implant Digital Impressions: A Systematic Review and Meta-Analysis

**DOI:** 10.1055/s-0045-1806935

**Published:** 2025-05-01

**Authors:** Saurabh Jain, Huda Ali Daak, Lena Abdulrahman Someli, Amwaj Yahya Alamer, Abhishek Apratim, Ruaa Mohammed Ali Akoor, Mohammed Ayoub

**Affiliations:** 1 Department of Prosthetic Dental Sciences, College of Dentistry, Jazan University, Jazan, Saudi Arabia; 2 College of Dentistry, Jazan University, Jazan, Saudi Arabia; 3 Mount Royal Dental, Mt. Forest Dr, Burlington, Ontario, Canada

**Keywords:** dental implants, digital impression, stereophotogrammetry, PIC dental, ICam4D, intraoral scanner, accuracy

## Abstract

This systematic literature review aimed to evaluate the accuracy of the stereophotogrammetry based dental scanners in determining complete-arch implant retained prosthesis compared to intraoral scanners (IOSs). The focused PICO (Population, Intervention, Comparison, Outcome) directed was “Do complete arch implant (P) impressions made using stereophotogrammetry-based dental scanners (I) have the same accuracy (O) when compared to impressions made using IOS (C)?” Recommendations listed by the Preferred Reporting Items for Systematic Reviews and Meta-Analyses (PRISMA) were used for structuring and reporting this review. This systematic review and meta-analysis was preregistered in the International Prospective Register of Systematic Reviews (PROSPERO) bearing the registration number CRD42024597913. To search the relevant titles, four electronic databases (MEDLINE/PubMed, Scopus, Cochrane Library, and Web of Science) were systematically searched in October 2024. The inclusion criteria include research papers published up to September 2024 in English comparing the accuracy of stereophotogrammetry-based dental scanners with IOS in recording the impression of complete-arch implants. Studies conducted on animals were excluded. Also excluded were unpublished reports, theses and dissertations, and case reports. After the initial search of the selected databases, a total of 590 titles were identified. The synthesis included 13 articles for qualitative analysis, but only 8 provided comparative data for quantitative analysis, which was performed using review manager (RevMan) Version 5.4. in non-Cochrane mode. The Modified CONSORT scale was used for *in vitro* quality and risk-of-bias assessment, while the QUADAS-2 tool was utilized for *in vivo* studies. The systematic review and meta-analysis reveals that stereophotogrammetric-based dental scanners offer higher accuracy in recording complete-arch implant-supported prosthesis impressions compared to IOS. The current review and meta-analysis compared of the accuracy of stereophotogrammetry-based dental scanners with IOSs. Limitations include medium to high quality of selected studies, with most of the *in vitro* studies displaying a high risk of bias, high heterogeneity in the control groups, and generalizability concerns. Accuracy of dental implant impressions is influenced by the type of scanner used for scanning. Stereophotogrammetry-based dental scanners are more accurate than IOS.

## Objectives

The application of advanced technologies in dentistry is helping dentists provide the best possible treatment to patients with predictable and consistent long-term clinical outcomes, 1 2 compared to conventional techniques, which are prone to human errors. 3 4 5 Implant-supported prosthesis is a standard treatment modality for rehabilitating patients with few or all missing teeth. 6 Passive fit is the key to success for a full-mouth implant prosthesis, as it prevents bone resorption and fracture of implant components in the long term. 7 8 9 10 11 12 Successful recording of the implant positions is amongst some of the critical steps in fabricating such prostheses. Much documented research data favor using digital technologies to make impressions for implant-retained prostheses. 6 8 11 12 Digital impression-making involves either digitalizing the impression or master cast with the help of extra-oral/bench scanners or making direct digital impressions using intraoral scanners (IOSs). The digital scanners should have high accuracy to ensure the final prosthesis has a passive fit. The currently available IOSs are based on different technologies. Parallel confocal scanning technology is used by Trios (3Shape) and iTero (Align Technology), while Primescan (Dentsply) uses dynamic depth scan technology. In contrast, CEREC Omnicam (Dentsply) uses the triangulation technique, and the True Definition scanner (3M ESPE) is based on active wavefront technology. 13 14 15 Studies have reported a high accuracy of IOSs compared to conventional techniques using impression materials in making an impression of implant-supported prostheses. For a passive fit of prosthesis, the acceptable threshold of deviations ranges between 10 and 50 microns, 9 10 11 12 whereas for a complete arch implant-supported prosthesis, this may range between 50 and 150 microns. 12 The use of IOSs for recording complete arch implant prosthesis impressions is reported to have some inherent shortcomings. These may be (1) operator-related, which includes scanning strategy, 16 operator experience; (2) implant-related: implant location, number of implants, inter-implant distance, 17 implant angulation 18 19 20 ; (3) patient-related: arch length, 17 mouth opening; and (4) technology-related: type of IOS, small wand size of IOS, 12 21 22 23 24 25 stitching concept for acquiring data and building up the image, 12 21 22 23 24 25 ambient light conditions, 26 27 type of scan body material, 19 presence or absence of reference points, and software-related. 25 28 29 Despite inherent shortcomings, using IOSs makes workflow more efficient, consumes less time, and is more patient-compliant. 30 31 Digital data allow immediate evaluation and decrease lab-related steps, thus reducing human errors. 30 31 Constant advancements in these IOSs are being made to overcome this shortcoming.

The introduction of stereophotogrammetry (SPG) in the field of dentistry in 1999 revolutionized the impression-making procedure, especially in complete arch implant prosthesis scenarios. 3 32 SPG is an extra-oral-based scanning technique in which two stereo cameras are placed at a predefined distance extra-orally to simultaneously detect the specific flag-shaped scan bodies attached to the dental implants. This technique records, measures, and interprets photographs without any physical contact with the scan bodies. Reference points in the photographs are used for locating implant positions and making precise measurements. 3 13 31 33 34 35 The merits of SPG lie in the fact that it can be used in the presence of saliva or blood without affecting its precision. 36 The technique does not require constant movement as with IOSs, and thus the chances of incorporation of errors are minimal. Additionally, as two cameras simultaneously detect and record the scan bodies, there is no need for a stitching process, thus reducing the errors. 4 5 32 35 37 38 39 The PIC dental scanner is based on the SPG technique, where two infrared cameras record the position of polka-dot–based scan bodies positioned in the patient's mouth. Other cameras based on SPG include ICam4D (Imetric4D Imaging), Oxo Fit (Oxo), and MicronMapper (S.I.N. Dental, United States). 4 5 37 38 39 There are multiple studies published in the literature comparing the accuracy of SPG-based dental scanners with conventional impressions and IOSs. Most studies have reported high accuracy of SPG dental scanners, but some reported conflicting results in recording impressions of complete-arch implant prosthesis. 3 13 19 30 40 41 42 43 44 45 46 47 48 49

There is a research gap in the literature that can quantitatively analyze the accuracy of SPG-based dental scanners in making an impression of a complete arch implant-retained prosthesis compared to IOS. To our knowledge, no published meta-analyses have compared this aspect. This systematic review and meta-analysis aimed to evaluate the accuracy of the SPG-based dental scanner in making an impression of a complete arch implant-retained prosthesis compared to IOS. The findings of this study may improve the understanding of the new technologies that can help the dentist provide high-quality treatment to their patients.

## Methods

### Eligibility Criteria

Recommendations listed by the Preferred Reporting Items for Systematic Reviews and Meta-Analyses (PRISMA) 50 51 were used for structuring and reporting this review. The inclusion criteria include research papers published up to September 2024 in English comparing the accuracy of SPG-based dental scanners with IOS in recording the impression of complete arch implants. Studies conducted on animals were excluded. Also excluded were unpublished reports, theses and dissertations, and case reports.

### Exposure and Outcome

The exposure in this study was the impression of complete arch implants made using IOS and SPG techniques. The outcome was the accuracy assessment of these two impression-making techniques. The focused PICO (Population, Intervention, Comparison, Outcome) directed was “Do complete arch implant (P) impressions made using SPG-based dental scanners (I) have the same accuracy (O) when compared to impressions made using IOS (C).”

### Search strategy, Study Selection, and Data Extraction

To search the relevant titles, two researchers independently used the above-mentioned PICO question and searched four electronic databases (MEDLINE/PubMed, Scopus, Cochrane Library, and Web of Science) on October 11 and 12, 2024. The key words and Boolean operators used are listed in Supplementary Table S1 . Minor alterations were made in the search string to meet the requirements of each database.

Duplicate titles were removed, and later, a third author matched and cross-checked the titles. Following this, two authors reviewed the titles and abstracts of these articles, and noneligible titles were excluded. Full texts of the remaining articles were reviewed, and the articles that met the set selection criteria were shortlisted for inclusion. A manual search of the references of the articles was conducted to make sure that no pertinent articles were left. Any conflicts between two authors related to the selection of articles were resolved after discussion with a third author. A self-designed table was used to extract relevant information, such as author, year, and country where the study was conducted, type of study, arch and model used, number of implants/analogues placed, reference group, comparator group, IOS and SPG & SB used, software used for superimposition, sample size, controlled ambient conditions, accuracy parameters tested, deviations/discrepancies reported, and author suggestions/conclusions ( Table 1 ).

**Table 1:** Summary of the studies included in the systematic review

Author, year, country	Study type	Arch and model used	No. of implants/analogues, company and placement angulation	Reference group	Comparator group/control IOS and SB	Study group/implant IOS/SPG and SB	Software for superimposition	Sample size/No. of scans	Controlled ambient conditions	Accuracy parameters tested Precision/trueness/LD/AD	Deviations/discrepancy	Author suggestions/conclusions
Tohme et al, 2021, Lebanon 3	In vitro	Acrylic resin edentulous maxillary model	4 implants (2 parallel anterior and 2 17° angled posterior) (Institut Straumann AG)	CI Digitalized using extra oral desktop scanner (E3; 3Shape A/S) SB: CARES Mono SB	IOS: TRIOS 3 (3Shape A/S) SB: Cares Mono SB (Institut Straumann A/S)	SPG: PiC camera (PiC dental) SB: PiC transfers	Geomagic Control X 2018; 3D Systems	*n* = 31 (control: 1 scan, study group: 15 scans per group)	Lighting: 1,003 lux PiC camera: placed 15 to 30 cm away from the cast Maximum angle = 45°	Trueness and precision	*Trueness:* 3D deviation (RMS in µm); IOS: 536 ± 63; SPG: 78 ± 1 *Angular deviation* (degree): IOS: 1.744 ± 0.175; SPG: 0.724 ± 0.064 *Precision:* 3D deviation (RMS in µm); IOS: 39 ± 9 SPG: 14 ± 13 *Angular deviation* (degree): IOS: 0.632; SPG: 0.947	Trueness (RMS and angular) PIC > TRIOS 3 Precision (RMS) PIC > TRIOS 3 Precision (angular) TRIOS 3 > PIC
Revilla-León et.al, 2021, United States 39	In vitro	Polymer resin edentulous maxillary model	6 implant analogs (two in canine region: 4° angled, two in first premolar region: 10° angled, two in first molar region: 0° angled) (Institute Straumann AG)	Digitalized definitive cast. CMM (CMM Contura G2 10/16/06 RDS; Carl Zeiss Industrielle Messtechnik GmbH): to measure the SB position	1) CI (Splinted impression copings) 2) IOS 1: iTero Element; Cadent 3) IOS 2: TRIOS 3 (3Shape A/S) SB: CARES Mono SB (Institut Straumann A/S)	SPG: ICam4D (Imetric4D Imaging Sàrl) SB: ICamBody (Imetric4D Imaging Sàrl)	Geomagic Control, 3D Systems	*n* = 30 (control: 10 scans per IOS, study group: 10 scans)	Lighting: 1,003 lux	Trueness and precision	1) Linear discrepancies (µm): A) *Trueness (median):* *x-* axis: CI: 7.3; SPG: 23.8; IOS1: 4.1; IOS2: 9.7 *y-* axis: CI: 8.9; SPG: 73.7; IOS1: 17.5; IOS2: 18 *z-* axis: CI: 1.8; SPG: −4.7; IOS1: −4.1; IOS2: −4.9 B) Precision (IQR) *x-* axis: CI: 17.9; SPG: 308.7; IOS1: 16.6; IOS2: 54.6 *y-* axis: CI: 17.5; SPG: 273.6; IOS1: 48.9; IOS2: 54.9 *z-* axis: CI: 4.6; SPG: 27.2; IOS1: 37.3; IOS2: 20.7 C) 3D discrepancy: CNV: 11.7; SPG: 77.6; IOS1: 18.4; IOS2: 21.1 2) Angular discrepancies (degrees) A) *Trueness (median):* *XZ angle:* CI: 0.1; SPG: 0.1; IOS1: −0.1; IOS2: −0.2 *YZ angle:* CI: −0.1; SPG: 0.3; IOS1: −0.1; IOS2: 0.2 B) *Precision (IQR)* *XZ angle:* CI: 0.4; SPG: 0.6; IOS1: 0.1; IOS2: 0.4 *YZ angle:* CI: 0.3; SPG: 0.6; IOS1: 0.3; IOS2: 0.4	Trueness and precision: (linear and angular discrepancy): CI > iTero Element IOS > TRIOS 3 IOS > ICam4D SPG 3D discrepancy: ICam4D SPG > TRIOS 3 IOS > iTero Element IOS > CI
Ma et al, 2021, China 45	In vitro	Maxillary Stone master cast	6 implant analogs (two in lateral region: 0° and 2° angled, two in first premolar region: 7° and 11° angled, two in first molar region: 9° and 7° angled) (Institute Straumann AG)	Digitalized master model using laboratory reference scanner (E4; 3Shape)	1) CI (Splinted impression copings) 2) IOS 2: TRIOS 3 (3Shape A/S) SB: CARES Mono SB (Institut Straumann A/S)	SPG: ICam4D (Imetric4D Imaging Sàrl) SB: ICamBody (Imetric4D Imaging Sàrl)	Geomagic control X, 3D Systems	*n* = 30 (control: 10 scans per group, study group: 10 scans)	NM	Trueness and Precision	Precision (mean RMS value in µm) CI: 29.72 IOS: 37.07 SPG: 2.32 Trueness (mean RMS value in µm) CI: 29.75 IOS: 43.78 SPG: 24.43	Trueness and precision: ICam4D SPG > CI > TRIOS 3 IOS
Sallorenzo and Gómez-Polo, 2022, Spain 41	In vitro	Acrylic resin edentulous maxilla	6 implant analogs Implant Protesis Dental, Spain 2 casts Parallel Implants Angulated implants (0°, 10°, and 20°)	Digitalized model using CMM (Global Evo 09.15.08, serial No. 906; Hexagon Manufacturing Intelligence)	TRIOS 3 (3Shape A/S) SB: Elos Accurate IO Scan (Elos Medtech AB)	1. PiC camera (PiC Dental, Spain) 2. SB: PiC transfer (PiC dental) 3. Camera position: 15–30 cm from model	Geomagic Control X (3D Systems, Rock Hill, South Carolina)	*n* = 40, 10 scans per cast and imaging system	Temp: 21.5°C Room lighting: 1,000 lux	Trueness Linear deviations and angular deviations Precision Linear deviations and angular deviations	Mean linear Error (in µm) a) Parallel implants: IOS: 100 ± 292 PIC: 20 ± 32 b) Angulated implants IOS: 23 ± 205 PIC: 10 ± 65 Mean angulation errors: (in degree) a) Parallel implants: IOS: 1.177 ± 0.474 PIC: 0.354 ± 0.280 b) Angulated implants IOS: 0.529 ± 0.841 PIC: 0.084 ± 0.246	Precision and trueness for linear and angular measurements: PIC > Trios 3 Precision and trueness affected by implant angulation significantly to varied extent in both the systems. LD and AD more in cast with parallel implants as compared to angulated implants PIC system, LD < clinically acceptable thresholds (100 µm and 0.40°)
Kosago et al, 2023, Thailand 13	In vitro	Acrylic resin edentulous mandible	5 dental implants (three parallel anterior and two 17° angled posterior) (Institut Straumann AG, Switzerland)	Extraoral scanner (E4 scanner)	1. CI and master cast digitalized using E4 EOS (Impression copings, Institut Straumann AG), 2. IOS: Trios 4 (3Shape) 3. IOS: iTero Element 2 (Align Technology, Tempe, Arizona), 4. IOS: Primescan (Dentsply-Sirona, York, Pennsylvania) SB: CARES Mono SB, PEEK/TAN, Institut Straumann AG	SPG: PIC camera 2.0 (PIC Dental, Madrid, Spain). SB: PIC transfers	Geomagic Control X 2020.1.1, 3D Systems	*n* = 30, 5 scans each per imaging system	NM	Precision and trueness	*Precision:* *Mean RMS deviation (µm)* CI: 49.40 ± 13.39 Trios 4: 19.39 ± 3.61 Primescan: 28.58 ± 8.03 iTero: 67.72 ± 7.18 PIC: 5.46 ± 1.10 *Trueness:* *Mean RMS deviation (µm)* CI: 141.86 ± 5.58 Trios 4: 52.14 ± 3.88 Primescan: 57.24 ± 2.05 iTero: 67.72 ± 7.18 PIC: 48.74 ± 1.80	Precision: PIC > Trios 4 > Primescan > Conventional > iTero Trueness: PIC > Trios 4> Primescan > iTero > Conventional
Tohme et al, 2023, Lebanon 40	In vitro	Acrylic resin edentulous maxillary model	4 implants (2 parallel anterior and two 17° angled posterior) (Institut Straumann AG)	CI and master cast digitalized using an extraoral scanner (E3; 3Shape A/S) Impression post: TANd for screw-retained; Institut Straumann AG)	IOS: TRIOS 3 (3Shape A/S) SB: CARES Mono SB	SPG: PiC with a stereoscopic camera (PiC camera; PiC Dental)	Geomagic Control X 2018; 3D Systems	*n* = 31 (control: 1 scan, study group: 15 scans per group)	Lighting: 1003 lux PiC camera: placed 15 to 30 cm away from the cast Maximum angle = 45°	Trueness and precision	*Trueness* *3D deviation (RMS in µm)* CI: 115 ± 37 Trios 3: 148 ± 61 PIC: 88 ± 6 *Angular deviation (degree)* CI: 0.922 ± 0.194 Trios 3: 1.081 ± 0.348 PIC: 0.809 ± 0.005 *Precision* *3D deviation (RMS in µm)* CI: 103 ± 24 Trios 3: 63 ± 35 PIC: 2 ± 1 *Angular deviation (degree)* CI: 1.142 ± 0.296 Trios 3: 0.221 ± 0.088 PIC: 0.010 ± 0.011	Trueness (3D deviation and angular) PIC > Conventional > Trios 3 Precision (3D deviation and angular) PIC > Trios 3 > Conventional
Pinto et al, 2024 Portugal 30	In vitro	Acrylic resin edentulous mandible	6 dental implants (Institut Straumann AG) and 4 dental implants	Industrial scanner (ATOS Capsule scanner; GOM GmbH) SB: Straumann CARES; Institut Straumann AG	1. EOS: D2000 (3Shape A/S) 2. EOS: S900 Arti (Zirkonzahn) 3. IOS: TRIOS 3 (3Shape A/S) 4. IOS: iTero Element 5D (Align Technology) SB: Straumann CARES; Institut Straumann	1) PIC Dental SB: PIC transfers 2) iCam (iMetric4D) SB: ICamBody; Imetric 4D Imaging Sàrl	Geomagic Control X (3D Systems, Rock Hill, South Carolina)	*n* = 144, 12 scans per cast and imaging system	NM	Precision	Precision error in RMS (µm) *6 implant scan* (mean ± CI) GOM: 2.23 (2.01; 2.45) D2000: 3.17 (3.01; 3.34) S900: 2.15 (2.04; 2.25) TRIOS 3: 40.32 (36.29; 44.36) iTero: 38.86 (34.01; 43.71) PIC: 13.88 (12.62; 15.14) ICAM: 8.67 (8.06; 9.28) *4 implant scan:* GOM: 1.67 (1.49; 1.86) D2000: 3.61 (3.23; 4.00) S900: 2.05 (1.89; 2.21) TRIOS 3: 20.52 (18.33; 22.72) iTero: 20.50 (17.37; 23.63) PIC: 5.18 (4.60; 5.76) ICAM: 7.01 (6.11; 7.91)	Overall precision • GOM > EOS > SPG > IOS • Precision for 6 implant case: • ICAM > PIC > ITero > Trios 3 • Precision for 4 implant case: • PIC > ICAM > ITero > Trios 3 • Precision: 6 implant scans < 4 implant scans Discrepancies more for posterior implants
Cheng et al, 2024, China 46	In vitro	Maxillary master model with resin soft tissue component and aerospace-grade aluminum alloy base	6 parallel implants corresponding to canines, second premolar, and second molar (bSKY4012, SKY)	Digitalized master model using industrial blue light scanner (ATOS Capsule 12 m, ATOS)	1) CI (splinted impression copings) Impression copings: Skyucaol, SKY 2) IOS 2: TRIOS 3 (3Shape A/S) without splinting SB: Universal SB (Segma) 3) IOS 2: TRIOS 3 (3Shape A/S) with splinting SB: Multi Unit SB (Segma)	SPG: ICam4D (Imetric4D Imaging Sàrl) SB: ICamBody (Imetric4D Imaging Sàrl)	Geomagic control X, 3D Systems	*n* = 40 (control: 10 scans per group, study group: 10 scans)	NM	Trueness, precision, linear, and angular measurement	RMS 3D deviation (µm): Trueness: CI: 87.3 IOS: 79.6 MIOS: 89.5 SPG: 69.2 Precision: CI: 77.1 IOS: 59.2 MIOS: 73.4 SPG: 41.1	Trueness: ICam4D SPG > IOS > CI > MIOS Precision: ICam4D SPG > IOS > MIOS > CI Adding a splint to the SB did not improve intraoral scanning accuracy
Pozzi et al 2024, Italy 31	In vitro	Acrylic resin edentulous mandibular model	4 multiunit implant analogs (MUA analogs; Nobel Biocare, Kloten, Switzerland) Angulation Anterior: 5° and 0° Posterior: 10° and 15°	D2000, 3 shape, Copenhagen, Denmark (Blue LED camera, scanner)	IOS: iTero Element 5D (Align Technology, Tempe, AZ, United States) SB: PEEK ISBs	SPG: PiC camera (PiC Dental, Madrid, Spain) SB: PIC Transfers	Geomagic Studio 12, 3D Systems, Rock Hill, South Carolina, United States	*n* = 61 (control: 1 scan, study group: 30 scans per group)	PiC camera position: 15–30 cm from the model 45° angulation	Linear deviations 3D mean deviations angular deviations	1. Linear deviations (µm) IOS: Δ *X* : 5.21 ± 50.51 Δ *Y* : −2.03 ± 14.54 Δ *Z* : −1.85 ± 37.31 SPG: Δ *X* : −12.81 ± 19.23 Δ *Y* : 0.95 ± 7.15 Δ *Z* : 20.78 ± 20.42 2. 3D mean deviations: IOS: 52.8 ± 37.11µm SPG: 33.42 ± 17.71 µm 3. Angular deviations: IOS: 0.28 ± 0.14 degree SPG: 0.24 ± 0.04 degree	Linear, 3D, and angular Accuracy PIC > iTero Element 5D IOS scanning revealed higher extreme deviations exceeding the acceptable threshold value. SPG more feasible than IOS for complete-arch digital implant impressions.
Orejas-Perez et al, 2022, Spain 42	*In vivo*	One patient with 8 implants in each arch	8 implants each in maxillary and mandibular arch (Medical Precision Implants MPI, Madrid, Spain)	Pre- established limits of misfit between the abutments and their angulations: a limit of 75 µm per segment and 0.6 degrees	IOS: Trios 3 (3Shape A/S Copenhagen, Denmark) SB: Elos Accurate 6A-B SB IOS: True Definition, (Midmark, Midmark Co., United States)	SPG: PiC camera; PiC dental) (Iditech North West SL)	Geomagic (Geomagic Inc., 3D Systems)	*n* = 30 scans (5 scans per arch, per scanner)	NM	*Precision* Linear error	Mean linear error (µm) Trios 3: 59.76 ± 44.51 True definition: 46.73 ± 37.02 PIC: 29.24 ± 20.65	Precision: PIC > True definition > Trios 3 The increase in the distance between implants affected the precision of both tested IOSs but not the PIC system
Fu et al, 2024, China 43	*In vivo*	22 edentulous arches 9 maxillary and 13 mandibular arches	Patients with 4–6 implants 4 ( *n* = 3), 5 ( *n* = 11), 6 ( *n* = 8) implants NobelActive, Nobel Biocare AB	CI digitized by scanning final casts with lab scanner (T8, Medit Corp.) Multiunit abutment analogs	IOS: TRIOS 3, 3Shape A/S SB: unique titanium SB	SPG: PIC camera (PIC Dental) SB: PIC Transfers	Geomagic Studio 2014, 3D Systems	*n* = 66, 22 scans per group	PiC camera position: 15–30 cm from the patient's mouth	Angle deviation RMS errors	Median angle deviation when compared to CI: IOS: 0.40° SPG: 0.31° RMS error compared to CI: IOS: 69 µm SPG: 45 µm Chairside time in minutes: IOS: 10.49 ± 3.50 SPG: 14.71 ± 2.86 CI: 20.20 ± 3.01	Accuracy PIC > TRIOS 3 Accuracy of OIC and TRIOS 3 clinically comparable. Efficiency of workflow TRIOS 3 > PIC > CI
Pozzi et al 2023, Italy 44	*In vivo*	11 edentulous arches 5 maxillary, 6 mandibular arches in 11 patients	Patients with 4 ( *n* = 8) And 6 ( *n* = 3) implants NobelActive, NobelParallel; NobelBiocare AG	CI digitalized with a high- resolution laboratory scanner (D2000; 3Shape)	IOS: Trios 4; 3Shape A/S SB: Elos Accurate Multi-Unit (Elos Medtech)	SPG: PiC camera (PiC dental) SB: PIC Transfers	Geomagic Studio 12; 3D Systems	*n* = 33, 11 scans per group	PiC camera position: 15–30 cm from the patient's mouth Angulation: 90° to 45°	● Linear deviations ● 3D deviations ● 3) Angular deviations	1. Linear deviations (µm): Overall mean IOS: 21.93 ± 100.60 SPG: 16.37 ± 63.91 2. 3D deviations (µm): IOS: 137.2 ± 115.5 SPG: 87.6 ± 74.2 3. Angular deviations (°): IOS: 0.79 ± 0.59 SPG: 0.38 ± 0.29	Linear and angular accuracy PIC > Trios 4 Higher extreme deviations noticed with Trios 4
Yan et.al, 2023, China 47	*In vivo*	Maxillary and mandibular jaw	4–8 implants (Bone Level Tapered Implant; Institute Straumann AG)	Digitalized definitive cast using high-resolution laboratory scanner (E4; 3Shape A/S)	IOS: CS3600; Carestream SB: CARES Mono SB for SRA (Institute Straumann AG)	SPG: ICam4D (Imetric4D Imaging Sàrl) SB: ICamBody (Imetric4D Imaging Sàrl)	Geomagic control X, 3D Systems	*n* = 194 (74 for IOS, 120 for SPG) No. of arches: IOS: 12; SPG:21	SPG placed approximately 20 cm away from the SB	3D deviation	*3D deviation (* µm) (median) IOS: 48.95 SPG: 17	Accuracy ICam4D SPG > IOS: CS3600

Abbreviations: #, data retrieved using Plot Digitizer App; AD, angular deviation; CI, confidence interval; CI, conventional impression using open tray technique and cast poured; CMM, coordinate measuring machine; EOS, extra-oral scanner; IOS, intraoral scanner; LD, linear deviation; NM, not mentioned; PIC, precise implant capture; PVS, polyvinyl siloxane; SB, scan bodies; SPG, stereophotogrammetry; Temp., temperature.

### Quality and Risk of Bias Assessment of Included Studies

The assessment of *in vitro* studies was performed using the Modified CONSORT scale for *in vitro* studies 52 53 ( Supplementary Table S2 ), whereas for *in vivo* studies, the QUADAS-2 (Quality Assessment of Diagnostic Accuracy Studies) tool was used 54 ( Supplementary Table S3 ). The modified CONSORT tool comprises 15 items that help in evaluating the studies. The first three items (1, 2, 2a) are associated with the article summary, backgrounds, and objectives. The following eight items (3–10) are associated with methodology, with items 6 to 9 primarily focusing on randomization and blinding. Item 11 is associated with the results section, and the last three items (12, 13, and 14) are associated with trial limitations, research funding, and accessibility of complete trial procedures. The QUADAS-2 tool evaluates the risk of bias and applicability concerns. The four domains involved in the risk of bias section are patient selection, index test, reference standard, and flow and timing. Whereas the applicability section involves all but the flow and timing domains.

### Meta-Analysis

For quantitative analysis, review manager (RevMan) Version 5.4 (The Cochrane Collaboration, 2020) was used in non-Cochrane mode. 55 Different types of IOSs were compared with the SPG technique. An inverse variance was used to calculate the standardized mean difference using a fixed-effects model. Based on the kind of IOS used, further subgrouping was done. Chi-square was used to measure heterogeneity. The forest plots created showed the pooled results for each subgroup and the overall pooled standardized mean difference.

## Results

### Identification, Screening, and Study Selection

**Fig. 1:**
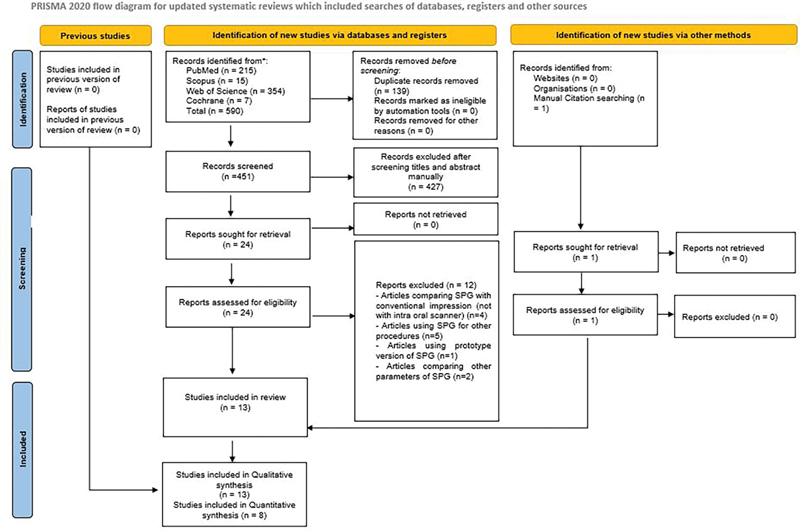
Article selection strategy based on PRISMA guidelines.

After the initial search of the selected databases, a total of 590 titles were identified. One additional article was found after a manual bibliographic search. One hundred and thirty-nine titles that were found to be duplicates were removed. Four hundred twenty-seven titles were removed after reviewing the titles and abstracts. Finally, 25 articles were reviewed and tested for eligibility based on pre-fixed selection criteria. Out of 25 articles, 4 articles compared SPG with conventional impression (not with IOS); 5 articles were related to the use of SPG for other procedures; 1 was using a prototype version of SPG; and 2 were comparing other parameters of SPG-based scanners. Any dispute between the reviewing authors was sought after a joint discussion with a third author. The end synthesis included 13 articles for qualitative analysis, out of which 8 were included in quantitative analysis ( Fig. 1 ).

### Quality Assessment of the Included Studies

Nine out of 13 studies included were *in vitro* studies. 3 13 30 31 39 40 41 45 46 Fifty-nine percent (80 out of 135) of the entries were positively documented. Items 1–5 and 10–12 were reported positively by all nine studies. Six studies reported Item 13 positively and two studies reported Item 14 positively. Whereas none of the studies reported items 6 to 9 positively, which were primarily related to randomization and blinding. For four *in vivo* studies, 42 43 44 47 the risk of bias was assessed using the QUADAS-2 tool. All four studies reported patient inclusion criteria, and all patients underwent impressions with both the tested scanners. So, the risk of bias in patient selection was low. As it is not clear whether the included studies interpreted the results without knowledge of the outcomes of the reference standard, the risk of bias was unclear for this domain. The reference standard domain displayed a low risk of bias. As all the patients underwent scanning by both test and control groups and there were no interventions between the index test and reference standard, this domain also displayed low risk. The applicability concern section also displayed similar results as per the risk of bias section.

### Study Characteristics

Nine of the 13 selected studies were *in vitro* , 3 13 30 31 39 40 41 45 46 and 4 were *in vivo* . 42 43 44 47 All the included studies were published in the last 5 years. Out of 13 studies, 3 were conducted in China 45 46 47 and 2 each were conducted in Lebanon, 3 40 Spain, 41 42 and Italy. 31 44 Similarly, one each was conducted in Thailand, 13 China, 43 United States, 39 and Portugal. 30

Seven out of nine *in vitro* studies were conducted on edentulous acrylic resin models, four using maxillary arch, 3 39 40 41 and the other three using mandibular arch. 13 30 31 One *in vitro* study used stone maxillary master cast, 45 whereas another study used a maxillary model with resin soft tissue component with aerospace-grade aluminum alloy base. 46 Both arches were involved in all the *in vivo* studies. The number of implants/analogues placed and later scanned varied from four, 3 31 40 43 44 47 five, 13 43 six, 30 39 43 44 45 46 47 and eight. 42 47 In some studies, parallel implants were placed 41 46 ; in some, implants were placed at an angulation, 42 whereas few had a combination of both. 3 13 31 39 40 45 The IOSs used for comparison varied among the studies. Commonly used IOSs were Trios 3, 3 30 39 40 41 42 43 45 46 Trios 4, 13 44 iTero 2, 13 CS3600 Carestream, 47 and iTero 5. 30 31 All the studies used the same software (Geomagic Studio) to superimpose the images and calculate the deviation. The properties compared were linear deviations, trueness, and precision in terms of 3D (three-dimensional) deviation and angular deviation.

### Studies Analyzing the Linear Deviations

**Fig. 2:**
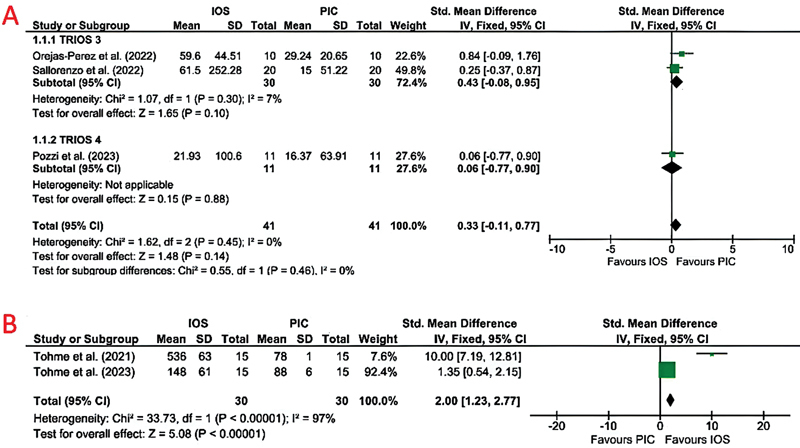
( **A** ) Forest plot comparing the linear deviation values of IOS and stereophotogrammetry technique. ( **B** ) Forest plot comparing the trueness in terms of 3D deviation values of IOS and stereophotogrammetry technique. 3D, three-dimensional; IOS, intraoral scanners.

Four studies compared the accuracy of IOS with a SPG-based dental scanner in terms of linear deviations. 39 41 42 44 Three studies used SPG-based PIC dental scanner, 41 42 44 whereas, one study used the ICam4D SPG scanner. 39 Three studies used Trios 3, 39 41 42 while one used Trios 4. 44 All the three studies using PIC dental scanner reported higher linear deviations when impressions were made with IOS scanners compared to PIC dental scanners. Whereas, when ICam4D was compared to IOS, the linear deviations reported were higher for ICam4D. 39 Due to heterogeneous nature, the study involving ICam4D scanner 39 was not included in the quantitative analysis. Trios 4 reported a lower linear deviation (21.92 ± 100.6) 44 as compared to Trios 3 (59.76 ± 44.51 and 61.5 ± 252.28). 41 42 There was no heterogeneity between the subgroups (I ^2^ = 0%), and the subgroup difference was statistically not significant ( *p* = 0.46). There was no statistically significant heterogeneity between the studies, with I ^2^ = 0%. The results were inconclusive, favoring the PIC dental scanner ( *p* = 0.14; Fig. 2A ).

### Studies Analyzing the Trueness in Terms of 3D Deviations

Two studies compared the trueness of IOS with SPG-based dental scanners in terms of 3D deviations. 3 40 45 46 47 Two studies used SPG-based PIC dental scanner, 3 40 whereas, three studies used the ICam4D SPG scanner. 45 46 47 The IOS used was Trios 3 by four studies, 3 40 46 47 whereas, one study used CS3600 IOS. Both the studies using PIC Dental scanner reported higher 3D deviations when impressions were made with IOS (536 ± 63 and 148 ± 61) compared to PIC dental scanners (78 ± 1 and 88 ± 6). Studies using ICam4D also reported higher 3D deviations for IOS (79.6, 43.78, and 48.95) when compared to ICam4D scanner (69.2, 24.43, and 17). Due to heterogeneous nature, the study involving ICam4D scanners 45 46 47 was not included in the quantitative analysis. There was statistically significant heterogeneity between the studies, with I ^2^ = 97%. The pooled estimate favored the PIC dental scanner with *p* < 0.00001 ( Fig. 2B ).

### Studies Analyzing the Trueness in Terms of Angular Deviations

**Fig. 3:**
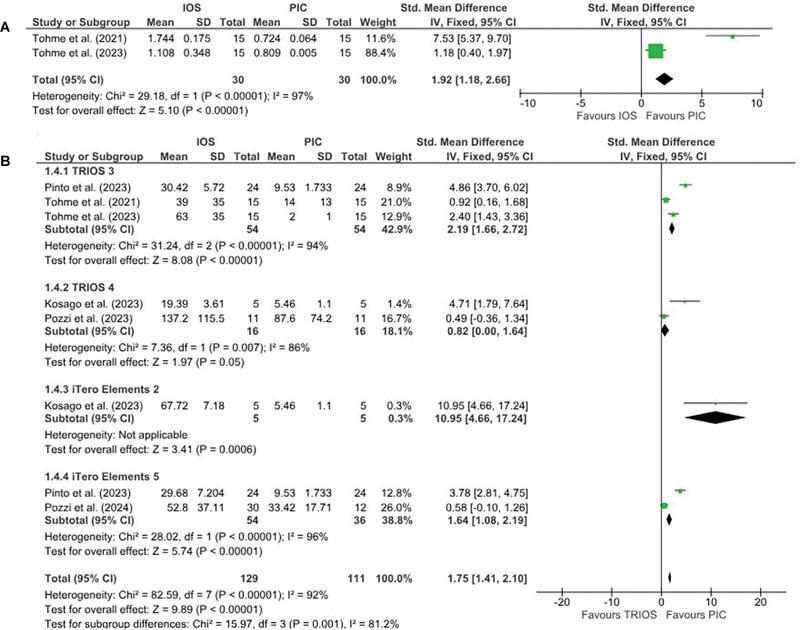
( **A** ) Forest plot comparing the trueness in terms of angular deviation values of IOS and stereophotogrammetry technique. ( **B** ) Forest plot comparing the precision in terms of 3D deviation values of IOS and stereophotogrammetry technique. 3D, three-dimensional; IOS, intraoral scanners.

Three studies compared the trueness of IOS with SPG-based dental scanners in terms of angular deviations. 3 39 40 Two studies used PIC scanner, 3 40 whereas one study used the ICam4D SPG scanner. 39 The IOS used was Trios 3 by all three studies. Study using the ICam4D SPG scanner reported higher angular deviations for SPG scanner when compared to IOS scanner. Whereas, both the studies using PIC dental scanner reported higher angular deviations when impressions were made with IOS scanners (1.744 ± 0.175 and 1.108 ± 0.348) compared to PIC dental scanners (0.724 ± 0.064 and 0.809 ± 0.005). Due to heterogeneous nature, the study involving ICam4D scanner 39 was not included in the quantitative analysis. There was statistically significant heterogeneity between the studies, with I ^2^ = 97%. Thus, the pooled estimate favored the PIC dental scanner with *p* < 0.00001 ( Fig. 3A ).

### Studies Analyzing the Precision in Terms of 3D Deviations

Eight studies compared the precision of IOS with a SPG-based dental scanner in terms of 3D deviations. Six of them used PIC Dental scanner, 3 13 30 31 40 44 whereas, two of them used ICam4D scanner. 45 46 Five of the studies used Trios 3, 3 30 40 45 46 two of them used Trios 4, 13 44 and iTero 5 30 31 each, while one used iTero 2. 13 All the studies reported higher 3D deviations when impressions were made with IOS scanners when compared to SPG-based dental scanners. Due to heterogeneous nature, the studies involving ICam4D scanner 45 46 were not included in the quantitative analysis. There was a statistically significant heterogeneity between the studies, with I ^2^ = 92%. Thus, the pooled estimate favored the PIC dental scanner with *p* = 0.001 ( Fig. 3B ). There was statistically significant heterogeneity between the subgroups ( *p* = 0.001).

### Studies Analyzing the Precision in Terms of Angular Deviations

**Fig. 4:**
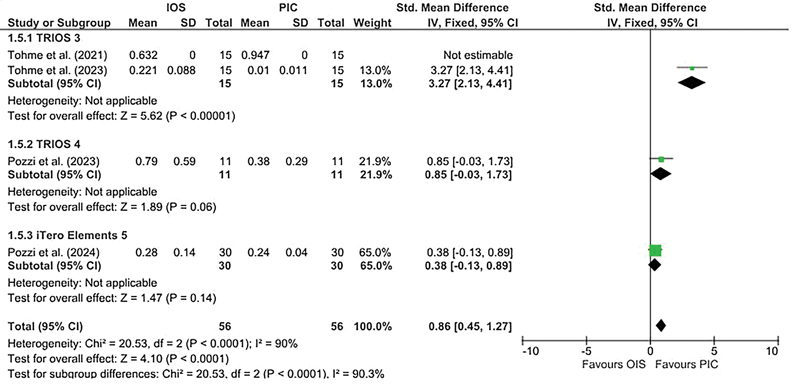
Forest plot comparing the precision in terms of angular deviation values of IOS and stereophotogrammetry technique. IOS, intraoral scanners.

Five studies compared the precision of IOS with a SPG-based dental scanner in terms of angular deviations. Four of them used PIC Dental scanner, 3 31 40 44 whereas one of them used ICam4D scanner. 39 Three of the studies used Trios 3, 3 39 40 while one each used Trios 4 44 and iTero 5. 31 All the studies using the PIC Dental scanner reported higher angular deviations when impressions were made with IOS scanners when compared to PIC dental scanners. However, the study using ICam4D scanner reported higher angular deviations with the SPG-based scanner. 39 Due to heterogeneous nature, the study involving ICam4D scanner 39 was not included in the quantitative analysis. There was a statistically significant heterogeneity between the studies, with I ^2^ = 90%. The pooled estimate favored the PIC dental scanner with *p* < 0.0001 ( Fig. 4 ). There was a statistically significant difference between the subgroups ( *p* < 0.0001).

## Discussion

The use of SPG-based dental cameras for recording implant positions has shown promising results. With multiple published studies comparing the accuracy of SPG-based dental scanners with other IOSs, it was deemed necessary to perform a thorough and organized analysis of these studies and deliver an updated summary of the available data. The focus was on analyzing the studies comparing the accuracy parameters of SPG-based dental scanners with other IOSs when recording impressions of full mouth implants. Thirteen articles were included in this systematic review, of which nine were *in vitro* studies, 3 13 30 31 39 40 41 45 46 and four were *in vivo* studies. 42 43 44 47 Due to the heterogeneous nature, five studies were not included in the meta-analysis. 39 43 45 46 47 A significantly higher accuracy of SPG-based scanners compared to IOSs was reported by this meta-analysis, which comprised eight studies conducted in different countries.

The included studies used various approaches to evaluate the accuracy (linear deviations, 3D dimensions, and angular deviations). Different schools of opinion exist regarding which approach is best for evaluating the accuracy. Supporters of 3D deviations state that this approach reports the root mean square deviation and there is no need to break it into various axes ( *x* , *y* , and *z* ) as this measuring coordinate system is different from the true coordinate system. Whereas some believe that it is crucial to know the direction of deviation ( *x* , *y* , and *z* -axes) as it is essential for the actual clinical fit of the implant-retained prosthesis. 13 42 For all the tested accuracy parameters, the SPG-based scanners demonstrated higher accuracies in recording impressions of full mouth implants when compared to IOS techniques. 3 13 30 31 40 41 42 43 44 45 46 47 Only one study reported lower accuracy of SPG-based scanners when compared to IOS. 39 The IOSs work on the best-fit algorithm-based process of stitching, in which software superimposes the consecutively captured 3D images through reproducible points. So, the higher the number of image stitches, the higher will be the errors. However, in SPG, the extra-oral camera has a larger field of view compared to IOSs and can identify and record all implants coordinated without stitching procedure. Thus, the errors related to stitching are not in SPG. 30 31 40 41 43 In two out of nine *in vitro* studies, the master model scanned with a benchtop scanner acted as the reference group, 13 31 45 in two studies conventional impressions were digitalized by scanning the master cast with an extra-oral scanner, 3 40 whereas in four studies the master cast was scanned using an industrial scanner. 30 39 41 46 Out of four *in vivo* studies, one used pre-established misfit limits as reference, 42 whereas the other three studies used conventional impressions digitalized by scanning master cast using an extra-oral scanner. 43 44 47 The following IOSs were used for comparison in most *in vitro* studies: Trios-3, 3 30 39 40 41 46 47 ITero Element 2 and 5, 13 30 31 39 Trios 4, 13 and Primescan. 13 In *in vivo* studies, the IOSs used were Trios 3, 42 Trios 4, 44 CS3600, 47 and True definition. 42 Some studies used a single IOS for comparison, while others used more than one IOS. 13 30 39 42 Direct comparison cannot be made between *in vitro* and *in vivo* studies, in general. For the SPG-based PIC scanner, *in vivo* studies reported higher 3D deviations for IOSs (137.2 µm ± 115.2) and PIC dental scanners (87.6 ± 74.2), 43 whereas for *in vitro* studies, the 3D deviations were reported to be in the range of 19.39 13 to 67.72 µm 13 for IOSs and 2 40 to 33.42 µm for PIC dental scanners. 13 Similar findings were reported for angular deviations also. *In vivo* studies reported higher angular deviation values for IOSs (0.79°), 43 whereas for *in vitro* the angular deviations ranged between 0.221° and 0.632°. For PIC dental scanners, these values were 0.38° for *in vivo* studies 43 and ranged between 0.01° 40 and 0.947° 3 for *in vitro* studies. For the SPG-based Icam4D scanner, the 3D deviation values for *in vitro* studies ranged from 24.43 45 to 77.6 µm. 39 Only one *in vivo* study using the ICam4D scanner reported a 3D deviation value of 17 µm. 47

Different studies documented different levels of clinically acceptable thresholds for a complete arch implant-supported prosthesis, with levels from 100 7 10 41 to 150 µm. 38 56 57 58 Angular deviation up to 0.40° of inter-implant deviation is considered a clinically acceptable level of error. 7 10 41 In all the included studies, the mean 3D deviation for SPG-based dental scanners and tested IOSs was well below this threshold level. However, one *in vivo* study 44 reported higher 3D deviation levels (137.2 µm ± 115) for IOS. One study reported higher angular deviations (0.947°) for PIC dental scanners when compared to IOS (0.632). 3 For other studies, 31 40 44 the angular deviations of SPG scanners were below the acceptable threshold levels and were less than those of IOSs. 56 57 58 59

The higher precision of SPG was reported to be because SPG, an extra-oral device, requires minimal movement initially to focus the scan bodies accurately, and later, it is kept stationary. In contrast, IOSs need to be moved by a trained clinician according to an appropriate scanning tactic to record all the scan bodies, thus making the technique sensitive. 31 59 Various other factors that provide an edge to SPGs in comparison to IOSs are the size of the scanner tip, saliva, scan body material, and ambient lighting. 30 31 37 39 59 60 61 A direct comparison between different tested IOS brands and generations cannot be made due to heterogeneity in the testing parameters of included studies. 60 61 62 Only one study reported lower accuracy of SPG scanners (ICam4D) when compared to IOSs. One study involved two different SPG scanners (PIC dental and ICam4D). 30 When used for scanning arch with six implants, a higher precision was reported for ICam4D scanners (8.67 µm) when compared to PIC scanner (13.88 µm). For scanning four implant arches, PIC scanners (5.18 µm) were reported to have higher precision than ICam4D scanners (7.01 µm).

Factors like number of implants, distance between implants, and implant angulations also play important roles in the accuracy of scanners. 10 12 62 Studies have reported that implant angulation within acceptable limits improves the accuracy of tested scanners. 3 41 Another parameter evaluated by one of the included studies was related to time taken to record the impressions. 43 They reported that the average time IOS took to scan one complete arch was 10.49 minutes, whereas SPG-based PIC dental scanners take 14.71 minutes and conventional impressions take 20.20 minutes for impression making. The SPG-based dental scanners have a few limitations, which include the necessity to use conventional IOSs in recording tissue surface, 13 40 limited applicability in patients with less mouth opening or small mouth, 13 40 and more time consumption in recording impressions, 43 especially when all the scan bodies are overlapping and cannot be recorded in one step.

A thorough search strategy, an organized methodology, and an unbiased evaluation of articles during study selection were the key highlights of this review. All studies comparing the accuracy of SPG-based scanners with IOSs were assessed to ensure that none of the pertinent studies were left out. Strength and limitations: while the outcomes of the current systematic review and meta-analysis add to a better understanding of the accuracy of SPG-based dental scanners when compared to IOSs, it is important to be cautious while interpreting them. This is due to some integral limitations of this review. The current review and meta-analysis were limited to the comparison of the accuracy of SPG-based dental scanners with IOSs. Comparisons with conventional impression techniques using elastomeric impression materials were not made. Other limitations include medium to high quality of selected studies, with most of the *in vitro* studies displaying a high risk of bias, high heterogeneity in the control groups, and generalizability concerns. More studies with standardized testing protocols and measuring techniques are required to make definite conclusions from meta-analysis regarding the linear deviations and trueness of SPG-based dental scanners.

## Conclusion

From the findings of the current systematic review and meta-analysis, it can be concluded that the SPG-based dental scanners have higher accuracy in recording impressions of complete-arch implant-supported prostheses when compared to IOS.
