# Supplementary material for: Accuracy of Stereophotogrammetry Technique versus Intraoral Scanners for Complete-Arch Implant Digital Impressions: A Systematic Review and Meta-Analysis
Source: Eur J Dent. 2025 May 1;20(1):34–48. doi: 10.1055/s-0045-1806935 (PMC12890421; doi:10.1055/s-0045-1806935)
Supplement: Supplementary file 1 — Supplementary Material [file 10-1055-s-0045-1806935-s24113922.pdf]

Supplementary Table S1 Search terms and strategy for the electronic databases

Database	Combination of search terms and strategy	Number of titles
MEDLINE PubMed	((‘Dental Implants*’ [MeSH] OR ‘Dental Prosthesis’ [MeSH] OR ‘Mouth, Edentulous*’ [MeSH] OR ‘Implant Supported Prosthesis’ [MeSH] OR ‘Models, Dental’ [MeSH] OR ‘Dental Implant’ OR ‘Complete Arch’ OR ‘Complete Dental Arch’ OR ‘Complete-Arch Fixed Dental Prostheses’ OR ‘implant-supported fixed prostheses’ OR ‘Implant Dentistry’ OR ‘Full mouth implant’ OR ‘Complete Arch Implant’ OR ‘Multiple Implant Restorations’)) AND (Photogrammetry [MeSH] OR Stereophotogrammetry OR ‘Extraoral Scanners’ OR PIC OR ‘PIC System’ OR ‘ICam4D’ OR ‘Implants Oral Scanner’ OR ‘PIC Dental’) AND (‘Dental Impression Technique’ [Mesh] OR ‘Intraoral Scanner’ OR ‘Digital Impression’ OR ‘Digital Scanning Systems’ OR ‘Implant Digital Scan’ OR ‘Implant Digital Impression’ OR ‘Implant Impression’ OR ‘Conventional Intraoral Scanner’) AND (‘Dimensional Measurement Accuracy’ [Mesh] OR Accuracy OR Trueness OR Precision OR ‘Linear Deviation’ OR Superimposition) AND (english[Filter]) AND ((humans [Filter]))	215
Web of Science (Core Collection)	#1 (P)<br>(TS = (‘Dental Implants*’ OR ‘Dental Prosthesis’ OR ‘Mouth, Edentulous*’ OR ‘Implant Supported Prosthesis’ OR ‘Models, Dental’ OR ‘Dental Implant’ OR ‘Complete Arch’ OR ‘Complete Dental Arch’ OR ‘Complete-Arch Fixed Dental Prostheses’ OR ‘implant-supported fixed prostheses’ OR ‘Implant Dentistry’ OR ‘Full mouth implant’ OR ‘Complete Arch Implant’ OR ‘Multiple Implant Restorations’))<br>#2 (I)<br>(TS= (Photogrammetry [MeSH] OR Stereophotogrammetry OR ‘Extraoral Scanners’ OR PIC OR ‘PIC System’ OR ‘ICam4D’ OR ‘Implants Oral Scanner’ OR ‘PIC Dental’))<br>#3 (C)<br>(TS= (‘Dental Impression Technique’ [Mesh] OR ‘Intraoral Scanner’ OR ‘Digital Impression’ OR ‘Digital Scanning Systems’ OR ‘Implant Digital Scan’ OR ‘Implant Digital Impression’ OR ‘Implant Impression’ OR ‘Conventional Intraoral Scanner’))<br>#4 (O)<br>(TS= (‘Dimensional Measurement Accuracy’ [Mesh] OR Accuracy OR Trueness OR Precision OR ‘Linear Deviation’ OR Superimposition))<br>#4 AND#3AND#2AND#1<br>Indexes = SCI-EXPANDED, SSCI, A&HCI, CPCI-S, CPCI-SSH, ESCI, CCR-EXPANDED, IC Time-span = All years AND LANGUAGE: (English)	354
Scopus	(“Dental Implant” OR “Dental Prosthesis” OR “Implant Supported Prosthesis” OR “Full mouth implant”) AND (“Photogrammetry” OR “Stereophotogrammetry” OR “PIC System” OR ‘ICam4D’ OR “Implants Oral Scanner”) AND (“Intraoral Scanner” OR “Digital Impression”) AND (“Accuracy” OR “Trueness” OR “Precision” OR “Linear Deviation”) AND (LIMIT-TO (DOCTYPE, “ar”) OR LIMIT-TO (DOCTYPE, “cp”)) AND (LIMIT-TO (SUBJAREA, “DENT”)) AND (LIMIT-TO (LANGUAGE, “English”)) AND (LIMIT-TO (SRCTYPE, “j”) OR LIMIT-TO (SRCTYPE, “p”))	15
Cochrane Library	ID Search Hits<br>#1 MeSH descriptor: [Dental Implants] explode all trees; #2 MeSH descriptor: [Dental Prosthesis] explode all trees; #3 MeSH descriptor: [Mouth, Edentulous] explode all trees; #4 MeSH descriptor: [Dental Prosthesis, Implant-Supported] explode all trees; #5 MeSH descriptor: [Models, Dental] explode all trees; #6 Complete Arch; #7 Complete Dental Arch; #8 Complete-Arch Fixed Dental Prostheses; #9 implant-supported fixed prostheses; #10 Implant Dentistry; #11 Full mouth implant; #12 Complete Arch Implant; #13 Multiple Implant Restorations; #14 MeSH descriptor: [Photogrammetry] explode all trees; #15Stereophotogrammetry; #16 Extraoral Scanners; #17 PIC System; #18 PIC Dental; #19 Implants Oral Scanner; #20 PIC; #21 ICam4D #22 MeSH descriptor: [Dental Impression Technique] explode all trees; #23 Intraoral Scanner; #24 Digital Impression; #25 Digital Scanning Systems; #26 Implant Digital Scan; #27 Implant Impression; #28 Conventional Intraoral Scanner; #29 MeSH descriptor: [Dimensional Measurement Accuracy] explode all trees; #30 Accuracy; #31 Trueness; #32 Precision; #33 Linear deviation; #34 Superimposition; #35 #1 OR #2 OR #3 OR #4 OR #5 OR #6 OR #7 OR #8 OR #9 OR #10 OR #11 OR #12 OR #13; #36 #14 OR #15 OR #16 OR #17 OR #18 OR #19 OR #20 OR #21; #37 #22 OR #23 OR #24 OR #25 OR #26 OR #27 OR #28; #38 #29 OR #30 OR #31 OR #32 OR #33 OR #34; #39 #35 AND #36 AND #37 AND #38 with ’Oral Health’, ’Methodology’ in Cochrane Groups	7

 © 2025. The Author(s).

Supplementary Table S2 Quality analysis results of the included studies performed using modified CONSORT scale for *in vitro* studies

Item →<br>Studies ↓	1	2a	2b	3	4	5	6	7	8	9	10	11	12	13	14
Tohme et al, 2021[3]	Y	Y	Y	Y	Y	Y	N	N	N	N	Y	Y	Y	Y	N
Revilla-León et al, 2021[39]	Y	Y	Y	Y	Y	Y	N	N	N	N	Y	Y	Y	N	N
Ma et al, 2021[45]	Y	Y	Y	Y	Y	Y	N	N	N	N	Y	Y	Y	Y	Y
Sallorenzo et al, 2022[41]	Y	Y	Y	Y	Y	Y	N	N	N	N	Y	Y	Y	Y	N
Kosago et al, 2023[13]	Y	Y	Y	Y	Y	Y	N	N	N	N	Y	Y	Y	Y	N
Tohme et al, 2023[40]	Y	Y	Y	Y	Y	Y	N	N	N	N	Y	Y	Y	Y	N
Pinto et al, 2023[30]	Y	Y	Y	Y	Y	Y	N	N	N	N	Y	Y	Y	N	N
Cheng et al, 2024[46]	Y	Y	Y	Y	Y	Y	N	N	N	N	Y	Y	Y	Y	Y
Pozzi et al, 2024[31]	Y	Y	Y	Y	Y	Y	N	N	N	N	Y	Y	Y	N	N

Supplementary Table S3 Quality assessment (QUADAS-2) summary of risk of bias and applicability concerns

Study	Risk of bias				Applicability concerns		
	Patient selection	Index test	Reference standard	Flow and timing	Patient selection	Index test	Reference standard
Orejas-Perez et al, 2022[42]	☺	?	☺	☺	☺	?	☺
Fu et al, 2023[43]	☺	?	☺	☺	☺	?	☺
Pozzi et al, 2023[44]	☺	?	☺	☺	☺	?	☺
Yan et al, 2023[47]	☺	?	☺	☺	☺	?	☺

Note: Levels of risk: ☺ low risk; ☹ high risk; ? unclear risk.

 © 2025. The Author(s).
